# Uncertainty Evaluation for the Quantification of Urinary Amphetamine and 4-Hydroxyamphetamine Using Liquid Chromatography–Tandem Mass Spectrometry: Comparison of the Guide to the Expression of Uncertainty in Measurement Approach and the Monte Carlo Method with R

**DOI:** 10.3390/molecules28196803

**Published:** 2023-09-25

**Authors:** Seon Yeong Kim, Dong Won Shin, Jihye Hyun, Nam Hee Kwon, Jae Chul Cheong, Ki-Jung Paeng, Jooyoung Lee, Jin Young Kim

**Affiliations:** 1 Forensic Genetics & Chemistry Division, Supreme Prosecutors’ Office, Seoul 06590, Republic of Korea; 2 Department of Applied Statistics, Chung-Ang University, Seoul 06590, Republic of Korea; zeze111111@cau.ac.kr; 3 Department of Chemistry, Yonsei University, Wonju 26493, Republic of Korea

**Keywords:** measurement uncertainty, Guide to the Expression of Uncertainty in Measurement, Monte Carlo method, amphetamine, LC–MS/MS

## Abstract

Estimating the measurement uncertainty (MU) is becoming increasingly mandatory in analytical toxicology. This study evaluates the uncertainty in the quantitative determination of urinary amphetamine (AP) and 4-hydroxyamphetamine (4HA) using a liquid chromatography–tandem mass spectrometry (LC–MS/MS) method based on the dilute-and-shoot approach. Urine sample dilution, preparation of calibrators, calibration curve, and method repeatability were identified as the sources of uncertainty. To evaluate the MU, the Guide to the Expression of Uncertainty in Measurement (GUM) approach and the Monte Carlo method (MCM) were compared using the R programming language. The MCM afforded a smaller coverage interval for both AP (94.83, 104.74) and 4HA (10.52, 12.14) than that produced by the GUM (AP (92.06, 107.41) and 4HA (10.21, 12.45)). The GUM approach offers an underestimated coverage interval for Type A evaluation, whereas the MCM provides an exact coverage interval under an abnormal probability distribution of the measurand. The MCM is useful in complex settings where the measurand is combined with numerous distributions because it is generated from the uncertainties of input quantities based on the propagation of the distribution. Therefore, the MCM is more practical than the GUM for evaluating the MU of urinary AP and 4HA concentrations using LC–MS/MS.

## 1. Introduction

Amphetamine (AP) is an active metabolite of methamphetamine and is a commonly prescribed drug for treating attention-deficit hyperactivity disorder (ADHD) [1,2]. ADHD diagnoses have recently increased, along with the use of prescription psychostimulants, particularly prescription ADHD medications [3,4]. Simultaneously, the abuse of these drugs has also increased among students globally, including in the Republic of Korea [5,6,7,8]. According to the Korean Ministry of Food and Drug Safety, the widespread use of ADHD psychostimulants among teenagers constitutes an epidemic that may contribute to future drug addictions [9]. Therefore, the Korean government has officially registered AP as a controlled substance owing to its high abuse potential. Although AP is less potent than methamphetamine, it is still a stimulant of the central nervous system. It is metabolized to benzoic acid, 1-phenylpropan-2-one, and 4-hydroxyamphetamine (4HA). 4HA, a sympathomimetic amine, is an active metabolite [4,5]. The presence of AP and 4HA in biological samples is generally determined using two types of hyphenated mass spectrometric methods: gas chromatography–mass spectrometry [6] and liquid chromatography–tandem mass spectrometry (LC–MS/MS) [7,8,10]. Herein, LC–MS/MS was used because it does not require an additional derivatization process for polar functional groups in target analytes [9], resulting in short sample preparation times.

The ISO/IEC 17025:2017 standard specifies the guidelines that enable laboratories to verify their competency and generate valid results, which promotes confidence in their analytical experiments [11]. Estimating the measurement uncertainty (MU) is being increasingly mandated in analytical toxicology by quality management standards, e.g., ISO/IEC 17025, to produce reliable results [12]. The Guide to the Expression of Uncertainty in Measurement (GUM) approach estimates the overall uncertainty based on the law of propagation of uncertainty or a bottom-up approach, identifying and quantifying the uncertainties in individual sources [13]. The GUM approach mainly involves specification, identification, quantification, and combination [14]. It provides a fundamental structure for evaluating the MU by assuming that all systematic errors are identical and amendable in the early stages of the evaluation. The GUM approves the use of its own approach and the Monte Carlo method (MCM) for the expression of the MU. However, the GUM approach has two main limitations. First, the first-order derivative of each component of the output quantity needs to be calculated, which requires mathematical expertise, particularly if the mathematical model is complex [15]. Second, it cannot exactly predict the probability distribution of the output quantity if the input quantities are not normally distributed. The MCM involves the propagation of the entire probability distribution of the input quantities without the need for calculating the first-order derivatives. Thus, it provides a numerical approximation to the distribution associated with a measurand, which is correlated with the measurement model and the distributions assigned to the input quantities [16]. It also provides a probability density function (PDF) for the output quantity as the final result, from which the coverage interval can be determined.

This study aims to estimate the MU associated with the quantification of urinary AP and 4HA using LC–MS/MS. Moreover, it examines the differences between the GUM approach and the MCM for calculating the MU of hardness and the input correlation effect on the uncertainty budget.

## 2. Results and Discussion

### 2.1. GUM Approach for Evaluating the Measurement Uncertainty

#### 2.1.1. Urine Sample Dilution

The urine sample (100 μL) was prepared by mixing it with an internal standard (IS) (50 μL) and mobile phase A (50 μL) before performing the LC–MS/MS analysis. The uncertainty associated with urine sample dilution, ${u(D}_{s})$, combines those resulting from the inaccuracies stemming from the use of a measuring pipette for urine sample dilution for sample preparation. The volume MU from pipetting was indicated in the calibration report as (100 ± 0.1) µL and (50 ± 0.1) µL with a coverage factor of 2. The standard uncertainties were evaluated as ${u(V}_{p100})=0.05\;µL$ and ${u(V}_{p50})=0.05\;µL$. The relative standard uncertainties were $u_{r}\left(V_{p100}\right)=0.0005$ and $u_{r}\left(V_{p50}\right)=0.001$:

(1) $$u_{r}(V_{p100})=\frac{u(V_{p100})}{V_{100}}\;(V_{100}=100\;\mu L),$$

(2) $$u_{r}(V_{p50})=\frac{u(V_{p50})}{V_{50}}\;(V_{50}=50\;\mu L).$$

The relative standard uncertainty ($u_{r}(D_{s})$) was 0.000866:

(3) $$u_{r}\left(D_{s}\right)=\sqrt{{\left(\frac{1}{2}\right)}^{2}\left\{{\left(\frac{u\left(V_{p100}\right)}{V_{p100}}\right)}^{2}+\frac{2{u\left(V_{p50}\right)}^{2}}{{\left({2V}_{p50}\right)}^{2}}\right\}}.$$

#### 2.1.2. Preparation of Calibrators

The uncertainty associated with the preparation of calibrators is approximated to the highest individual uncertainty of the preparatory steps—the MU of the reference standard and inaccuracy of the measuring devices (e.g., volumetric pipettes and volumetric flasks). The reference standards are used as calibrators and quality control (QC) samples in forensic toxicology [17]. The uncertainties of the certified ampoule solution reference standards of AP and 4HA were both (1.00 ± 0.006) mg/mL with a coverage factor of 2. The standard uncertainties were evaluated as ${u(C}_{R.AP})=0.003\;mg/mL$ and ${u(C}_{R.4HA})=0.003\;mg/mL.$ The relative standard uncertainties were $u_{r}\left(C_{R.AP}\right)=0.003$ and $u_{r}\left(C_{R.4HA}\right)=0.003$, as shown below:

(4) $$u_{r}\left(C_{R}\right)=\frac{u\left(C_{R}\right)}{C_{R}}(C_{R}=1.0\;mg/mL).$$

The volume MU from pipetting, ${u(V}_{p})$, was indicated in the calibration report as (1000 ± 1.5) µL and (500 ± 1.5) µL with a coverage factor of 2. The standard uncertainties were evaluated as ${u(V}_{p1000})=0.75µL$ and ${u(V}_{p500})=0.75µL$. The relative standard uncertainties were $u_{r}\left(V_{p1000}\right)=0.00075$ and $u_{r}\left(V_{p500}\right)=0.0015$:

(5) $$u_{r}(V_{p1000})=\frac{u(V_{p1000})}{V_{1000}}\;(V_{1000}=1000\;\mu L),$$

(6) $$u_{r}(V_{p500})=\frac{u(V_{p500})}{V_{500}}\;(V_{500}=500\;\mu L).$$

The volume MU from using a 10 mL glass volumetric flask, ${u(V}_{f})$, was indicated on the calibration report as (10 ± 0.006) mL with a coverage factor of 2. The standard uncertainty was evaluated as $u\left(V_{f10}\right)=0.003\;mL$. Additional uncertainty due to temperature differences between calibration time and the analysis time was estimated as ±5 °C, by considering the cubic thermal expansion coefficient of methanol (α = 0.00149 mL/°C) [18]. The temperature variation for the measurement step was (25 ± 5) °C, determined using a uniform distribution (Table 1) [13]. The standard uncertainty (${u(V}_{Tf})$) was 0.004301 mL:

(7) $$u\left(V_{Tf}\right)=\alpha \times V_{f10}\times u\left(T_{f}\right)=\frac{0.00149\times 10\times 5}{\sqrt{3}}\;(V_{f10}=10\;mL).$$

These standard uncertainty components are combined as one standard uncertainty according to the law of the propagation of errors. The combined standard uncertainty, ${u(V}_{f})$, was 0.043114 mL, and the estimated relative standard uncertainty ($u_{r}(V_{f})$) was 0.004311 mL:

(8) $$u\left(V_{f}\right)=\sqrt{{u\left(V_{f10}\right)}^{2}+{u\left(V_{Tf}\right)}^{2}},$$

(9) $$u_{r}\left(V_{f}\right)=\frac{u\left(V_{f}\right)}{V_{f}}(V_{f}=10\;mL).$$

The derivation of the uncertainty of calibrators is described in detail in Supplementary Materials S1. It includes the following steps: (1) preparation of the working standard solution; (2) preparation of the calibration standard solutions; and (3) dilution. The uncertainty associated with the preparation of the working standard solution ($u_{r}\left(W_{AP}\right)=0.006878$ and $u_{r}\left(W_{4HA}\right)=0.006878$) was calculated by combining the uncertainties resulting from the use of reference standard, pipettes, and volumetric flasks. The calibration standard solutions were obtained by serial dilution of the working standard solution with the pooled blank urine [19]. The uncertainty associated with the calibrators ($u_{r}\left(C_{CS.AP}\right)=0.018953$ and $u_{r}\left(C_{CS.4HA}\right)=0.019036$) was calculated by combining the uncertainties of pipettes, calibration standard solutions, and dilutions, as follows:

(10) $$u_{r}\left(C_{CS.AP}\right)=\sqrt{\sum_{i=1}^{7}{u_{r}\left(C_{i.AP}\right)}^{2}+{7\left(\frac{1}{2}\right)}^{2}\left\{{\left(\frac{u\left(V_{p100}\right)}{V_{p100}}\right)}^{2}+\frac{{2u\left(V_{p50}\right)}^{2}}{{\left(2V_{p50}\right)}^{2}}\right\}},$$

(11) $$u_{r}\left(C_{CS.4HA}\right)=\sqrt{\sum_{i=2}^{8}{u_{r}\left(C_{i.4HA}\right)}^{2}+{7\left(\frac{1}{2}\right)}^{2}\left\{{\left(\frac{u\left(V_{p100}\right)}{V_{p100}}\right)}^{2}+\frac{{2u\left(V_{p50}\right)}^{2}}{{\left(2V_{p50}\right)}^{2}}\right\}},$$

where $u_{r}\left(C_{i}\right)$ is the uncertainty of each calibration standard solution $C_{i}$ for i=1,…, 8.

#### 2.1.3. Calibration Curve

Calibration curves were prepared by plotting the peak area ratio against the analyte concentration, which required the preparation of calibrators (*n* = 7, see Section 3). These calibration curves were used to estimate the analyte concentrations in the urine samples. The calibration functions were calculated and fitted by a linear regression model with a weighting factor ($w_{i}$) yielding the following equation:

(12) $$y_{s}=a_{w}+b_{w}\times x_{s}$$

$$(y_{s}=\frac{A_{a}}{A_{is}},\;a_{w}=\frac{\sum (w_{i}\cdot x_{i}^{2})\cdot \sum {(w}_{i}\cdot y_{i})-\sum \left(w_{i}\cdot x_{i})\cdot \sum {(w}_{i}\cdot x_{i}\cdot y_{i}\right)}{\sum w_{i}\cdot \sum (w_{i}\cdot x_{i}^{2})-{(\sum \left(w_{i}\cdot x_{i})\right)}^{2}},\;b_{w}=\frac{\sum w_{i}\cdot \sum {(w}_{i}\cdot x_{i}\cdot y_{i})-\sum \left(w_{i}\cdot x_{i})\cdot \sum {(w}_{i}\cdot y_{i}\right)}{\sum w_{i}\cdot \sum (w_{i}\cdot x_{i}^{2})-{(\sum \left(w_{i}\cdot x_{i})\right)}^{2}},\;w_{i}=\frac{1}{x_{i}^{2}}),$$

where $x_{s}$ is a value on the *x*-axis and $y_{s}$ is the peak area ratio of the analyte ($A_{a}$) to its IS ($A_{is}$) in the urine sample [20].

The urine sample of an AP abuser was analyzed, and $x_{s}=99.74\;ng/mL$ for AP and $x_{s}=11.33\;ng/mL$ for 4HA were obtained. The standard uncertainties and relative standard uncertainties of AP and 4HA were determined by solving regression equations for the weighted model. The standard uncertainties of the calibration curve (${u(C}_{c})$) for AP and 4HA were 2.539030 and 0.372684, respectively, while their relative standard uncertainties ($u_{r}(C_{c})$) were 0.025458 and 0.032891, respectively, calculated as follows:

(13) $${u(C}_{c})=\frac{S_{w}}{b_{w}}\sqrt{\frac{1}{w_{s}}+\frac{1}{\sum w_{i}}+\frac{{\left(y_{i}-{\bar{y}}_{w}\right)}^{2}}{{b_{w}}^{2}\cdot \sum w_{i}{\left(x_{i}-{\bar{x}}_{w}\right)}^{2}}},$$

(14) $$u_{r}(C_{c})=\frac{u(C_{c})}{x_{s}}$$

$$(S_{w}=\sqrt{\frac{{\sum {(w}_{i}\cdot (y_{i}-(a_{w}+b_{w}\cdot x_{i}))}^{2}}{n-2}},\;{\bar{x}}_{w}=\frac{\sum \left(w_{i}\cdot x_{i}\right)}{\sum w_{i}},\;{\bar{y}}_{w}=\frac{\sum \left(w_{i}\cdot y_{i}\right)}{\sum w_{i}},\;x_{s}=\frac{\left(y_{s}-a_{w}\right)}{b_{w}}),$$

where $x_{s}$ is the determined concentration of the analyte, $y_{s}$ is the peak area ratio of the analyte, and $w_{s}$ is the weighting factor of the calibration curve in the urine sample.

#### 2.1.4. Method Repeatability

Method repeatability is estimated by conducting measurements in multiple independent experimental assays. To ensure the repeatability of the analytical methodology, QC samples were prepared for AP (30, 150, and 300 ng/mL) and 4HA (6, 30, and 150 ng/mL) in blank urine. Six independent determinations were performed on each prepared QC sample on four different days to obtain a repeatability estimate using the abovementioned method (Table 2). The relative standard uncertainties ($u_{r}(R_{qc})$) were 0.019355 for AP and 0.028505 for 4HA.

(15) $${u(R}_{qc})=\sqrt{\frac{\sum s_{i}^{2}\times (n_{i}-1)}{\sum \left(n_{i}-1\right)}},$$

(16) $$u_{r}\left(R_{qc}\right)=\frac{{u(R}_{qc})}{{\bar{x}}_{i}},$$

where $s_{i}$ is the standard deviation for multiple replicates, $n_{i}$ is the number of replicates, and ${\bar{x}}_{i}$ is the mean of the $n_{i}$ measurements [21]. Next, the relative standard uncertainty was linearly interpolated to estimate the corresponding value with the estimated measurand concentrations of AP and 4HA [22].

The effective degree of freedom ($v_{eff}$) was obtained by calculating the degree of freedom for each component of uncertainty using the Welch–Satterthwaite equation (Table 3):

(17) $$v_{eff}=\frac{u_{c}^{4}}{\sum \frac{u_{i}^{4}}{v_{i}}},$$

where $v_{i}$ is the degree of freedom of the *i*-th component of uncertainty, $u_{i}$ is the standard uncertainty of the *i*-th component, and $u_{C}$ is the combined uncertainty [23].

#### 2.1.5. Calculating the Combined and Expanded Uncertainty

The uncertainties from four individual components in the urine analysis of AP and 4HA were quantified (Table 3). The combined standard MU of the overall analytical method ($u_{C}$) was calculated as follows:

(18) $$u_{C}=\sqrt{{u_{r}\left(D_{s}\right)}^{2}+{{u_{r}\left(C_{CS}\right)}^{2}+u_{r}\left(C_{c}\right)}^{2}+{u_{r}\left(R_{qc}\right)}^{2}}.$$

If the measured quantity is related to the *t*-distribution from which the values are taken, the selection of *k* = 2.07 and 2.08 for AP and 4HA, respectively, is indicative of a 95% confidence level. Expanded uncertainty (U) was obtained by multiplying the combined standard MU ($u_{C})$ with the coverage factor (k), as shown below:

(19) $$U=k\times u_{c}=k\times C_{s}\times u_{rC},$$

(20) $$U_{r}=k\times u_{rC}.$$

**Figure 1:**
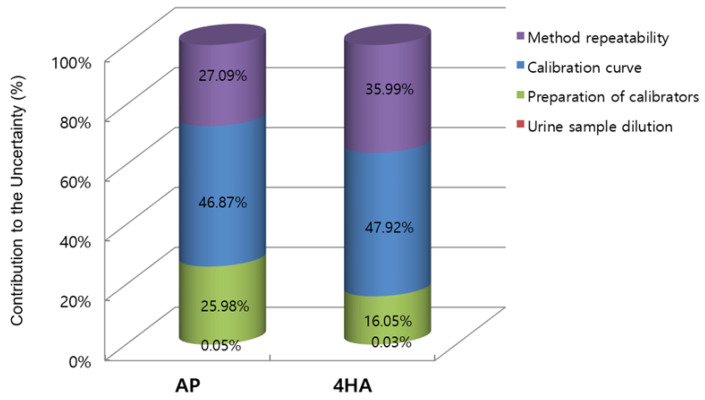
Relative contribution of the four different uncertainty sources to the overall combined uncertainty of amphetamine (AP) and 4-hydroxyamphetamine (4HA) in the Guide to the Expression of Uncertainty in Measurement (GUM) approach.

The expanded uncertainties of AP and 4HA in the sample were 7.68 and 1.12 ng/mL, respectively. Therefore, the concentrations of AP and 4HA in the real urine sample with their expanded uncertainties were 99.74 ± 7.68 and 11.33 ± 1.12 ng/mL, respectively. Figure 1 shows the relative contribution of the main uncertainty sources to the overall combined standard uncertainty for the quantification of urinary AP and 4HA using the GUM approach.

### 2.2. MCM for the Evaluation of MU

#### 2.2.1. Mathematical Modeling of the Measurand

The following mathematical modeling of the measurand was used for the MCM:

(21) $$C_{x}=C_{s}+\delta_{D}+\delta_{R},$$

where $C_{s}=\left(\frac{y_{s}-a_{w}}{b_{w}}\right)$, $y_{s}=\frac{A_{a}}{A_{is}}$, $y_{s}$ is the peak area ratio of the analyte $(A_{a}$*)* to its deuterated IS *(*$A_{is})$, $a_{w}$ is the y-intercept, $b_{w}$ is the calibration curve slope, $\delta_{D}$ is the additive factor for the urine sample dilution uncertainty, and $\delta_{R}$ is the additive factor for the method repeatability uncertainty.

The measurand for Equation (21) does not include the additive factor for the uncertainty of calibrators and calibration curve uncertainty because the calibration curve was estimated using error-contaminated calibrators (because of the uncertainty of calibrators). Therefore, $C_{s}$ indicates the randomness associated with the preparation of calibrators and the calibration curve. The PDFs of the reference standard ($C_{R}$), pipette ($V_{p}$), and 10 mL volumetric flask ($V_{f10}$) values were followed by the calibration report, whilethe volumetric expansion is assumed, as the temperature ($V_{Tf}$) has a uniform distribution (Table 1).

#### 2.2.2. MCM Simulation

The MCM algorithm was implemented in R version 4.0.1 with 10^6^ simulation trials (M = 10^6^). The calibrators were first generated by utilizing the serial dilution described in Supplementary Data S1. The uncertainty of response *y_i_* was obtained from the standard deviations of four repeated measured peak area ratios $\left(\frac{A_{a}}{A_{is}}\right)$ at each calibrator, while response *y_i_* was drawn from a normal distribution. The calibration curve was fitted using the weighted linear regression model with the generated calibrators. The responses of the peak area ratios and predicted measurand were obtained for each trial. The additive factors accounting for method repeatability and urine sample dilution were drawn from normal distributions with a mean of 0 and standard deviations obtained through the GUM approach, respectively. The R code to implement the MCM is available at Supplementary Data S2.

#### 2.2.3. Uncertainty with MCM and Comparison with GUM Approach

**Figure 2:**
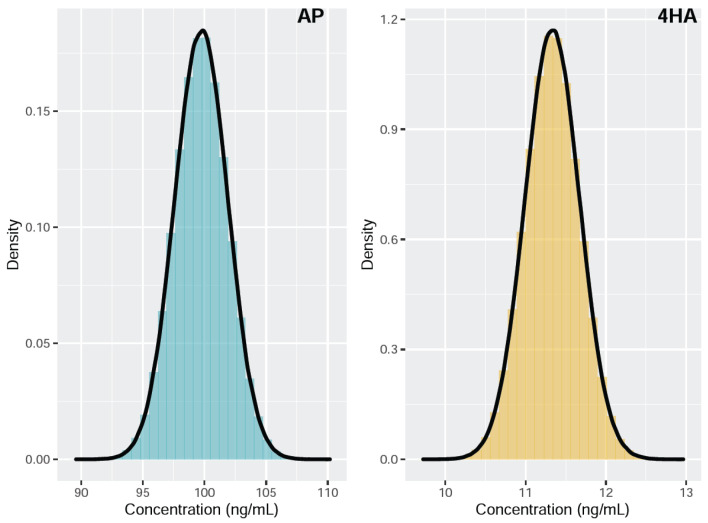
Probability density function of the concentration measurand for amphetamine (AP) and 4-hydroxyamphetamine (4HA).

Figure 2 shows the histograms overlaid with the kernel density estimates of the simulated measurand concentrations of AP and 4HA. The estimated AP concentration was 99.75 ng/mL, with an associated standard uncertainty of 2.53 ng/mL and a 95% coverage interval (94.83, 104.74). The estimated 4HA concentration was 11.33 ng/mL, with an associated standard uncertainty of 0.41 ng/mL and a 95% coverage interval (10.52, 12.14). The MCM provided a smaller coverage interval for both AP and 4HA compared to that obtained with the GUM approach (Table 4). In addition, the GUM approach underestimates the coverage interval, whereas the MCM provides an exact coverage interval under an abnormal probability distribution of the measurand [24]. Therefore, MCM is considered a more practical approach for evaluating MUs in this study.

In the GUM approach, the standard uncertainty is obtained by combining uncertainties for each error-accompanied factor (law of propagation of uncertainty), while the expanded uncertainty is calculated by multiplying a coverage factor corresponding to the 95% confidence level. However, the GUM approach has two limitations: (1) the measured values should follow a *t*-distribution to obtain the expanded uncertainties and (2) the uncertainty of calibrators and calibration curve uncertainty shown in Equation (18) should be additively combined. However, the intercept ($a_{w})$ and slope $\left(b_{w}\right)$ estimates in Equation (12) include calibrators, demonstrating a non-linear relationship between the calibrators and the measurand. Thus, simply adding the uncertainty of calibrators would be inaccurate; therefore, a correct uncertainty equation based on the law of propagation of uncertainty should be considered for the GUM approach. In the MCM, the measurand is generated from the uncertainties of input quantities using the propagation of distribution. The propagation of distribution is useful in complex settings where the measurand combines numerous distributions and the distribution of the measurand is unknown [25,26].

The GUM approach, based on the law of propagation of uncertainty, should be used with caution when the model includes complex non-linear elements. The uncertainty of calibrators was added to the combined uncertainty of other sources in most previous studies [22,27] and was also used here. However, this approach becomes invalid when the measurand and calibrators have non-linear relationships. When the analyte concentration of the samples is estimated, the measurement error of calibrators is not considered in the GUM approach. If this measurement error model can be considered an additive Berkson error problem of the form $x_{i}=u_{i}+\epsilon_{i}$, where $x_{i}$ is the true calibrator, $u_{i}$ is the error-contaminated calibrator, and $\epsilon_{i}$ is the additive Berkson error term, then the intercept and slope estimators become unbiased in a simple linear regression model [28]. However, the true calibrators and error-contaminated calibrators may not have an additive Berkson error problem, which leads to a bias between the intercept and slope estimators. In the present study, the uncertainty of calibrators is small, which results in negligible bias in the estimate of the measurand. The analytical standard uncertainty of the analyte based on the multiple standard addition method was investigated in a previous paper [29], and it accounted for the measurement error in the response and calibrators. Further research is required to account for the measurement error in the response and calibrators in an IS calibration method using the method of measurement error correction to obtain correct estimators and standard uncertainty.

## 3. Materials and Methods

### 3.1. Chemicals and Reagents

AP, 4HA, and AP-d_8_ were obtained as solutions from Cerilliant (Austin, TX, USA). HPLC-grade methanol was purchased from J.T. Baker/Avantor (Center Valley, PA, USA). Water (LiChrosolv-grade) was supplied by Merck (Darmstadt, Germany). Formic acid (LC–MS LiChropur-grade) was purchased from Sigma Aldrich (St. Louis, MO, USA). All other chemicals were of analytical grade or higher. The AP and 4HA working standard solutions (10 µg/mL) were prepared by dilution with methanol. The IS working solution was prepared in methanol to obtain an AP-d_8_ solution (0.1 µg/mL). These solutions were stored at −20 °C in amber bottles before use.

### 3.2. Preparation of Urine Sample

Blank urine samples were obtained from the laboratory staff with their consent. Pooled blank urine was used for preparing calibrators and QC samples. Forensic urine samples were obtained from the Narcotics Departments at the District Prosecutors’ Offices (Seoul Metropolitan Area). The urine sample (100 μL) was transferred to a 1.5 mL polypropylene tube (Eppendorf, Hamburg, Germany) and mixed with mobile phase A (50 μL) and IS (50 μL). After centrifugation at 50,000× *g* for 3 min, 8 μL of clear supernatant was injected into the LC–MS/MS system.

### 3.3. Preparation of Calibrators and QC Samples

The linearity of the method was evaluated over the concentration ranges for AP (5, 10, 25, 50, 100, 250, and 500 ng/mL) and 4HA (2, 5, 10, 25, 50, 100, and 250 ng/mL). The linear least-squares regression of the ratio of the analyte to the IS peak area, with a weighting factor of 1/*x*^2^, was used to generate a calibration curve. QC samples for AP (30, 150, and 300 ng/mL) and 4HA (6, 30, and 150 ng/mL) were prepared by spiking the pooled blank urine samples with known amounts of each compound to ensure the repeatability of the LC–MS/MS method. The inter-day precision of the method was established via six independent determinations with the same QC samples on four independent experimental assays of the abovementioned replicates (*n* = 24).

### 3.4. LC–MS/MS Conditions

LC–MS/MS was performed using a Shiseido (Osaka, Japan) Nanospace SI-2 HPLC system coupled to a Sciex (Foster City, CA, USA) QTRAP 6500 mass spectrometer. The target compounds were separated using a Hypersil GOLD C18 column (150 mm × 2.1 mm i.d., 5 μm, Thermo Scientific, Waltham, MA, USA). The mobile phases consisted of 0.4% formic acid in water (mobile phase A) and methanol (mobile phase B) at different ratios at a flow rate of 400 μL/min. Gradient elution was initiated with a solution containing 10% of mobile phase B for 1 min. The mobile phase B content was increased to 20% over 3.5 min, then to 90% over 7 min, and was then maintained at 90% for 1 min. Finally, the condition was changed to the initial composition (i.e., 10% of mobile phase B) for 3 min to stabilize the system. The column was thermostated at 35 °C, while the autosampler temperature was 10 °C. Electrospray ionization was performed in the positive mode. The ion spray voltage and ion source temperature were set at 5500 V and 600 °C, respectively. Ion source gases 1 and 2, curtain gas, and collision gas were set to 55, 80, 60, and medium (arbitrary units), respectively. The scheduled multiple-reaction monitoring (MRM) mode was operated with a 60 s detection window. The most abundant and specific ion transitions were selected as the quantitative ion pairs of the target compounds, while the second-most abundant ones were employed as the qualitative ion pairs. The MRM ion pairs were set as follows: *m/z* 136 → 91 and *m/z* 136 → 119 for AP, *m/z* 152 → 132 and *m/z* 152 → 107 for 4HA, and *m/z* 144 → 97 for AP-d_8_.

### 3.5. Identification of the Uncertainty Sources

An estimation of the MU associated with the analytical results can be obtained by considering different individual uncertainties. The main uncertainty sources associated with quantifying AP and 4HA in urine were urine sample dilution, preparation of calibrators, calibration curve, and method repeatability.

### 3.6. Uncertainty Estimation Method

The uncertainty was quantified using the GUM approach and the MCM. The MU was estimated using the GUM approach as follows: (1) the measurand was defined; (2) possible uncertainty factors and sources were identified; (3) their contribution to MU was estimated; (4) the combined uncertainties were calculated; and (5) the expanded uncertainty was expressed to define a probability range. The uncertainty was calculated using the MCM as follows: (1) the measurand was defined; (2) possible uncertainty factors and sources were identified; (3) their contribution to MU was estimated using PDF; (4) the number of simulations was selected; (5) the simulation procedure was conducted; and (6) the measurand probability range was expressed based on the probability density distribution.

### 3.7. Specifying the Measurand

The measurand was defined as the concentration of AP and 4HA in each urine sample and was expressed using Equation (22):

(22) $$C_{x}=C_{s}+\delta_{D}+\delta_{CS}+\delta_{C}+\delta_{R},$$

where $C_{x}$ is the concentration of AP or 4HA in the urine sample, $C_{s}$ is the amount of AP or 4HA in the given sample volume, $\delta_{D}$ is the additive factor for the urine sample dilution uncertainty, $\delta_{CS}$ is the additive factor for the calibrators uncertainty, $\delta_{C}$ is the additive factor for the calibration curve uncertainty, and $\delta_{R}$ is the additive factor for the method repeatability uncertainty.

## 4. Conclusions

Herein, we discussed the application of the GUM approach and the MCM for calculating the uncertainty generated while quantifying the urinary AP and 4HA using LC–MS/MS. The MCM provided a smaller coverage interval for both AP and 4HA compared to that obtained using the GUM approach. Since MCM provides an exact coverage interval under an abnormal probability distribution of the measurand, it is more practical for evaluating MU. The methods proposed herein can be widely applied to the MU evaluation of quantitative analyses in various fields, including forensic chemistry.

## Figures and Tables

**Table 1:** Information about input quantities of the model.

Quantity	Unit	Value	Standard Uncertainty	Probability Density Function
$C_{R}$	mg/mL	1.00	0.003	Normal (1.00, ${\left(0.003\right)}^{2}$)
$V_{f10}$	mL	10	0.003	Normal (10, ${\left(0.003\right)}^{2})$
$V_{Tf}$	°C	-	0.04301	Uniform (−0.0745, 0.0745)
$V_{p1000}$	μL	1000	0.75	Normal (1000, ${\left(0.75\right)}^{2}$)
$V_{p500}$	μL	500	0.75	Normal (500, ${\left(0.75\right)}^{2}$)
$V_{p100}$	μL	100	0.1	Normal (100, ${\left(0.1\right)}^{2}$)
$V_{p50}$	μL	50	0.1	Normal (50, ${\left(0.1\right)}^{2}$)

**Table 2:** Results of the evaluation of method repeatability from low-, middle-, and high-quality control samples.

Analyte	Nominal Concentration (ng/mL)	Experiments							
		Assay 1		Assay 2		Assay 3		Assay 4	
		Mean (*n* = 6)	Standard Deviation	Mean (*n* = 6)	Standard Deviation	Mean (*n* = 6)	Standard Deviation	Mean (*n* = 6)	Standard Deviation
AP	30	32.3	0.4176	32.9	0.4840	29.3	0.3014	31.5	0.8831
	150	165.4	2.7589	160.4	1.5858	144.5	4.3782	155.9	3.3410
	300	308.7	3.6577	314.9	5.2759	288.8	4.2205	303.4	2.7928
4HA	6	6.7	0.1797	6.6	0.1135	5.8	0.1940	6.5	0.2496
	30	32.9	0.4256	33.6	0.6306	30.2	0.6749	31.4	0.9422
	150	163.2	3.5016	163.2	2.2802	145.3	2.5725	151.3	3.1556

**Table 3:** Estimation of uncertainty contributions in quantitative LC–MS/MS analysis results of amphetamine (AP) and 4-hydroxyamphetamine (4HA) in urine.

Source of Uncertainty	AP			4HA		
	Relative Standard Uncertainty	Effective Degrees of Freedom	Degree of Contribution (%)	Relative Standard Uncertainty	Effective Degrees of Freedom	Degree of Contribution (%)
Urine sample dilution ($u_{r}(D_{S})$)	0.000866	∞	0.05	0.000866	∞	0.03
Preparation of calibrators ($u_{r}(C_{CS})$)	0.018953	∞	25.98	0.019036	∞	16.05
Calibration curve ($u_{r}(C_{C})$)	0.025458	5	46.87	0.032891	5	47.92
Method repeatability ($u_{r}(R_{qc})$)	0.019355	55	27.09	0.028505	51	35.99
Relative combined standard uncertainty ($u_{rc}(C)$)	0.037184	22	-	0.047512	21	-
Relative expanded uncertainty ($U_{r}(C)$)	0.076971	-	-	0.098826	-	-

**Table 4:** Statistical parameters obtained for the Guide to the Expression of Uncertainty in Measurement (GUM) approach and the Monte Carlo method (MCM) for the determination of urinary concentrations of amphetamine (AP) and 4-hydroxyamphetamine (4HA).

Statistical Data	AP		4HA	
	GUM	MCM	GUM	MCM
Mean (ng/mL)	99.74	99.75	11.33	11.33
Standard deviation (ng/mL)	3.71	2.53	0.54	0.41
95% confidence interval (ng/mL)	(92.06, 107.41)	(94.83, 104.74)	(10.21, 12.45)	(10.52, 12.14)

## Data Availability

The data presented in this study are available in manuscript and Supplementary Materials.

## Supplementary Materials

The following supporting information can be downloaded at https://www.mdpi.com/article/10.3390/molecules28196803/s1. The derivation for uncertainty of calibrators is provided in Supplementary Data S1. The R code for uncertainty evaluation with the MCM for the quantification of urinary AP and 4HA using LC–MS/MS is available Supplementary Data S2.

## Sample Availability

Samples of the compounds are not available from the authors.
