# Supplementary material for: Uncertainty Evaluation for the Quantification of Urinary Amphetamine and 4-Hydroxyamphetamine Using Liquid Chromatography–Tandem Mass Spectrometry: Comparison of the Guide to the Expression of Uncertainty in Measurement Approach and the Monte Carlo Method with R
Source: Molecules. 2023 Sep 25;28(19):6803. doi: 10.3390/molecules28196803 (PMC10574584; doi:10.3390/molecules28196803)
Supplement: Supplementary file 1 [file molecules-28-06803-s001.zip › Supplementary Data S1.pdf]

Derivation for uncertainty of calibrators

[1] Preparation of the working standard solution

1. W_S solution (100 µg/mL)

Add 1 mL of reference standard of AP to a 10 mL volumetric flask with methanol.

$$u_r(W_{S.AP}) = \sqrt{\left(\frac{u(W_S)}{W_S}\right)^2} = \sqrt{\left(\frac{u(C_{R.AP})}{C_{R.AP}}\right)^2 + \left(\frac{u(V_{p1000})}{V_{p1000}}\right)^2 + \left(\frac{u(V_f)}{V_f}\right)^2} = 0.005306$$

Add 1 mL of reference standard of 4HA to a 10 mL volumetric flask with methanol.

$$u_r(W_{S.4HA}) = \sqrt{\left(\frac{u(W_S)}{W_S}\right)^2} = \sqrt{\left(\frac{u(C_{R.4HA})}{C_{R.4HA}}\right)^2 + \left(\frac{u(V_{p1000})}{V_{p1000}}\right)^2 + \left(\frac{u(V_f)}{V_f}\right)^2} = 0.005306$$

2. W solution (10 µg/mL)

Add 1 mL of W_S solutions of AP and 4HA to a 10 mL volumetric flask with methanol.

$$u_r(W_{AP}) = \sqrt{\left(\frac{u(W_{AP})}{W_{AP}}\right)^2} = \sqrt{\left(\frac{u(W_{s.AP})}{W_{s.AP}}\right)^2 + \left(\frac{u(V_{p1000})}{V_{p1000}}\right)^2 + \left(\frac{u(V_f)}{V_f}\right)^2} = 0.006878$$

$$u_r(W_{4HA}) = \sqrt{\left(\frac{u(W_{4HA})}{W_{4HA}}\right)^2} = \sqrt{\left(\frac{u(W_{s.4HA})}{W_{s.4HA}}\right)^2 + \left(\frac{u(V_{p1000})}{V_{p1000}}\right)^2 + \left(\frac{u(V_f)}{V_f}\right)^2} = 0.006878$$

[2] Preparation of the calibration standard solutions

1. C_1 solution (1000 ng/mL)

Mix 0.5 mL of W solution and 9 × 0.5 mL of blank urine.

$$u_r(C_1) = \sqrt{\left(\frac{u(C_1)}{C_1}\right)^2} = \sqrt{\left(\frac{u(W)}{W}\right)^2 + \left(\frac{9}{10}\right)^2 \times \left\{\left(\frac{u(V_{p500})}{V_{p500}}\right)^2 + \frac{9u(V_{p500})^2}{(9V_{p500})^2}\right\}} = 0.007023$$

2. C_2 solution (500 ng/mL)

Mix 1.0 mL of C_1 solution and 1.0 mL of blank urine.

$$u_r(C_2) = \sqrt{\left(\frac{u(C_2)}{C_2}\right)^2} = \sqrt{\left(\frac{u(C_1)}{C_1}\right)^2 + \frac{1}{2}\left(\frac{u(V_{p1000})}{V_{p1000}}\right)^2} = 0.007043$$

3. C_3 solution (200 ng/mL)

Mix 2 × 0.5 mL of C_2 solution and 3 × 0.5 mL of blank urine.

$$u_r(C_3) = \sqrt{\left(\frac{u(C_3)}{C_3}\right)^2} = \sqrt{\left(\frac{u(C_2)}{C_2}\right)^2 + \left(\frac{1.5}{2.5}\right)^2 \left\{2\left(\frac{u\left(V_{p500}\right)}{2V_{p500}}\right)^2 + \frac{3u\left(V_{p500}\right)^2}{(3V_{p500})^2}\right\}} = 0.007091$$

4. C_4 solution (100 ng/mL)

Mix 1.0 mL of C_3 solution and 1.0 mL of blank urine.

$$u_r(C_4) = \sqrt{\left(\frac{u(C_4)}{C_4}\right)^2} = \sqrt{\left(\frac{u(C_3)}{C_3}\right)^2 + \frac{1}{2}\left(\frac{u\left(V_{p1000}\right)}{V_{p1000}}\right)^2} = 0.007111$$

5. C_5 solution (50 ng/mL)

Mix 1.0 mL of C_4 solution and 1.0 mL of blank urine.

$$u_r(C_5) = \sqrt{\left(\frac{u(C_5)}{C_5}\right)^2} = \sqrt{\left(\frac{u(C_4)}{C_4}\right)^2 + \frac{1}{2}\left(\frac{u\left(V_{p1000}\right)}{V_{p1000}}\right)^2} = 0.007131$$

6. C_6 solution (20 ng/mL)

Mix 2 × 0.5 mL of C_5 solution and 3 × 0.5 mL of blank urine.

$$u_r(C_6) = \sqrt{\left(\frac{u(C_6)}{C_6}\right)^2} = \sqrt{\left(\frac{u(C_5)}{C_5}\right)^2 + \left(\frac{1.5}{2.5}\right)^2 \left\{2\left(\frac{u\left(V_{p500}\right)}{2V_{p500}}\right)^2 + \frac{3u\left(V_{p500}\right)^2}{(3V_{p500})^2}\right\}} = 0.007178$$

7. C_7 solution (10 ng/mL)

Mix 1.0 mL of C_6 solution and 1.0 mL of blank urine.

$$u_r(C_7) = \sqrt{\left(\frac{u(C_7)}{C_7}\right)^2} = \sqrt{\left(\frac{u(C_6)}{C_6}\right)^2 + \frac{1}{2}\left(\frac{u\left(V_{p1000}\right)}{V_{p1000}}\right)^2} = 0.007197$$

8. C_8 solution (4 ng/mL)

Mix 2 × 0.5 mL of C_7 solution and 3 × 0.5 mL of blank urine.

$$u_r(C_8) = \sqrt{\left(\frac{u(C_8)}{C_8}\right)^2} = \sqrt{\left(\frac{u(C_7)}{C_7}\right)^2 + \left(\frac{1.5}{2.5}\right)^2 \left\{2\left(\frac{u\left(V_{p500}\right)}{2V_{p500}}\right)^2 + \frac{3u\left(V_{p500}\right)^2}{(3V_{p500})^2}\right\}} = 0.007244$$

[3] Dilution

Each calibration standard solution (100 μL) was mixed with mobile phase A (50 μL) and IS (50 μL) in a 1.5-mL polypropylene tube following the Preparation of Urine Sample step.

$$u_r(C_{CS.AP}) = \sqrt{\sum_{i=1}^{7} u_r(C_{i.AP})^2 + 7\left(\frac{1}{2}\right)^2 \left\{\left(\frac{u\left(V_{p100}\right)}{V_{p100}}\right)^2 + \frac{2u\left(V_{p50}\right)^2}{(2V_{p50})^2}\right\}} = 0.018953$$

$$u_r(C_{CS.4HA}) = \sqrt{\sum_{i=2}^{8} u_r(C_{i.4HA})^2 + 7\left(\frac{1}{2}\right)^2 \left\{\left(\frac{u\left(V_{p100}\right)}{V_{p100}}\right)^2 + \frac{2u\left(V_{p50}\right)^2}{(2V_{p50})^2}\right\}} = 0.019036$$
