# Supplementary material for: Uncertainty Evaluation for the Quantification of Urinary Amphetamine and 4-Hydroxyamphetamine Using Liquid Chromatography–Tandem Mass Spectrometry: Comparison of the Guide to the Expression of Uncertainty in Measurement Approach and the Monte Carlo Method with R
Source: Molecules. 2023 Sep 25;28(19):6803. doi: 10.3390/molecules28196803 (PMC10574584; doi:10.3390/molecules28196803)
Supplement: Supplementary file 1 [file molecules-28-06803-s001.zip › Supplementary Data S2.pdf]

[1] R code for calculation of uncertainty with MCM

```
# number of trials

M <- 10^6

# Parameters for the pdf of input quantities

uCr <- 0.003

uVp1000 <- 0.00075

uVp500 <- 0.00075

uVp100 <- 0.0001

uVp50 <- 0.0001

uVf10 <- 0.003

alpha  <- 0.00149

a <- 25 - 5*alpha*10

b <- 25 + 5*alpha*10

# generate the volume

Vp1 <- rnorm(M, 1, uVp1000)

Vp0.5 <- rnorm(M, 0.5, uVp500)

Vp0.1 <- rnorm(M, 0.1, uVp100)

Vp0.05 <- rnorm(M, 0.05, uVp50)

Temp <- runif(M, 20, 30)

Vf <- rnorm(M, 10, uVf10)*(1+alpha*(Temp-25)) # temperature effect

# generate the reference material

Cr <- rnorm(M, 1, uCr)

# initialize concentrations

x0AP <- rep(0, M)

x04HA <- rep(0, M)

# the peak area ratio of the analyte in the sample

y0.AP <- 0.2

y0.4HA <- 0.021
```

```
# generate working standard solution

W1 <- Cr*rnorm(M, 1, uVp1000)/(rnorm(M, 10, uVf10)*(1+alpha*(Temp-25)))

W <- W1*rnorm(M, 1, uVp1000)/(rnorm(M, 10, uVf10)*(1+alpha*(Temp-25)))

# generate calibration standard solutions

C1 <- W*rnorm(M, 0.5, uVp500)/(rnorm(M, 0.5, uVp500)+rnorm(M, 0.5, uVp500)+
                               rnorm(M, 0.5, uVp500)+rnorm(M, 0.5, uVp500)+
                               rnorm(M, 0.5, uVp500)+rnorm(M, 0.5, uVp500)+
                               rnorm(M, 0.5, uVp500)+rnorm(M, 0.5, uVp500)+
                               rnorm(M, 0.5, uVp500)+rnorm(M, 0.5, uVp500))

C2 <- C1*rnorm(M, 1, uVp1000)/(rnorm(M, 1, uVp1000)+rnorm(M, 1, uVp1000))

C3 <- C2*(rnorm(M, 0.5, uVp500)+rnorm(M, 0.5, uVp500))/(rnorm(M, 0.5, uVp500)+rnorm(M, 0.5,
uVp500)+rnorm(M, 0.5, uVp500)+rnorm(M, 0.5, uVp500)+rnorm(M, 0.5, uVp500))

C4 <- C3*rnorm(M, 1, uVp1000)/(rnorm(M, 1, uVp1000)+rnorm(M, 1, uVp1000))

C5 <- C4*rnorm(M, 1, uVp1000)/(rnorm(M, 1, uVp1000)+rnorm(M, 1, uVp1000))

C6 <- C5*(rnorm(M, 0.5, uVp500)+rnorm(M, 0.5, uVp500))/(rnorm(M, 0.5, uVp500)+rnorm(M, 0.5,
uVp500)+rnorm(M, 0.5, uVp500)+rnorm(M, 0.5, uVp500)+rnorm(M, 0.5, uVp500))

C7 <- C6*rnorm(M, 1, uVp1000)/(rnorm(M, 1, uVp1000)+rnorm(M, 1, uVp1000))

C8 <- C7*(rnorm(M, 0.5, uVp500)+rnorm(M, 0.5, uVp500))/(rnorm(M, 0.5, uVp500)+rnorm(M, 0.5,
uVp500)+rnorm(M, 0.5, uVp500)+rnorm(M, 0.5, uVp500)+rnorm(M, 0.5, uVp500))

# dilution of calibration standard solutions

X1 <- C1*rnorm(M, 0.1, uVp100)/(rnorm(M, 0.05, uVp50)+rnorm(M, 0.05, uVp50)+rnorm(M, 0.1, uVp100))

X2 <- C2*rnorm(M, 0.1, uVp100)/(rnorm(M, 0.05, uVp50)+rnorm(M, 0.05, uVp50)+rnorm(M, 0.1, uVp100))

X3 <- C3*rnorm(M, 0.1, uVp100)/(rnorm(M, 0.05, uVp50)+rnorm(M, 0.05, uVp50)+rnorm(M, 0.1, uVp100))

X4 <- C4*rnorm(M, 0.1, uVp100)/(rnorm(M, 0.05, uVp50)+rnorm(M, 0.05, uVp50)+rnorm(M, 0.1, uVp100))

X5 <- C5*rnorm(M, 0.1, uVp100)/(rnorm(M, 0.05, uVp50)+rnorm(M, 0.05, uVp50)+rnorm(M, 0.1, uVp100))

X6 <- C6*rnorm(M, 0.1, uVp100)/(rnorm(M, 0.05, uVp50)+rnorm(M, 0.05, uVp50)+rnorm(M, 0.1, uVp100))

X7 <- C7*rnorm(M, 0.1, uVp100)/(rnorm(M, 0.05, uVp50)+rnorm(M, 0.05, uVp50)+rnorm(M, 0.1, uVp100))

X8 <- C8*rnorm(M, 0.1, uVp100)/(rnorm(M, 0.05, uVp50)+rnorm(M, 0.05, uVp50)+rnorm(M, 0.1, uVp100))
```

```
# generate y based on the data for calibration curves
## dat.AP_cal: the data for calibration curves
## fit.AP: the fitted calibration curves: yi ~ a + b*xi + ei, var(ei) = (sigma*xi)^2
dat.AP_cal <- read_excel("~/Dropbox/2021_Research/검찰청/Output/calibration.xlsx",
    sheet = "AP")
dat.4HA_cal <- read_excel("~/Dropbox/2021_Research/검찰청/Output/calibration.xlsx",
    sheet = "4HA")
cal_AP_y <- read.csv("~/Dropbox/2021_Research/검찰청/Output/cal_AP_y.txt")

cal_4HA_y <-  read.csv("~/Dropbox/2021_Research/검찰청/Output/cal_4HA_y.txt")
y1 <- rnorm(M, dat.AP_cal$y[1], sqrt(cal_AP_y$var[1]))
y2 <- rnorm(M, dat.AP_cal$y[2], sqrt(cal_AP_y$var[2]))
y3 <- rnorm(M, dat.AP_cal$y[3], sqrt(cal_AP_y$var[3]))
y4 <- rnorm(M, dat.AP_cal$y[4], sqrt(cal_AP_y$var[4]))
y5 <- rnorm(M, dat.AP_cal$y[5], sqrt(cal_AP_y$var[5]))
y6 <- rnorm(M, dat.AP_cal$y[6], sqrt(cal_AP_y$var[6]))
y7 <- rnorm(M, dat.AP_cal$y[7], sqrt(cal_AP_y$var[7]))
for(i in 1:M){
  AP <- data.frame(x = 1000000*rev(c(X1[i], X2[i], X3[i], X4[i], X5[i], X6[i], X7[i])),
                   y= c(y1[i], y2[i], y3[i], y4[i], y5[i], y6[i], y7[i]))
  fit.AP <- lm(y~x, weight=1/x^2, data=AP)
  x0AP[i] <- (y0.AP - fit.AP$coefficients[1])/fit.AP$coefficients[2]
}
y1 <- rnorm(M, dat.4HA_cal$y[1], sqrt(cal_4HA_y$var[1]))
y2 <- rnorm(M, dat.4HA_cal$y[2], sqrt(cal_4HA_y$var[2]))
y3 <- rnorm(M, dat.4HA_cal$y[3], sqrt(cal_4HA_y$var[3]))
y4 <- rnorm(M, dat.4HA_cal$y[4], sqrt(cal_4HA_y$var[4]))
```

```
y5 <- rnorm(M, dat.4HA_cal$y[5], sqrt(cal_4HA_y$var[5]))

y6 <- rnorm(M, dat.4HA_cal$y[6], sqrt(cal_4HA_y$var[6]))

y7 <- rnorm(M, dat.4HA_cal$y[7], sqrt(cal_4HA_y$var[7]))

for(i in 1:M){

  HA <- data.frame(x = 1000000*rev(c(X2[i], X3[i], X4[i], X5[i], X6[i], X7[i], X8[i])),

                  y= c(y1[i], y2[i], y3[i], y4[i], y5[i], y6[i], y7[i]))

  fit.HA <- lm(y~x, weight=1/x^2, data=HA)

  x04HA[i] <- (y0.4HA - fit.HA$coefficients[1])/fit.HA$coefficients[2]

}

# relative uncertainty from the GUM

urD <- 0.000866

urR.AP <- 0.0193553

urR.4HA <- 0.0285052

# AP accounting for the uncertainty from method repeatability and urine sample dilution

x0AP.t <- x0AP + rnorm(M, 0, urR.AP*x0.AP) + rnorm(M, 0, urD*x0.AP)

#mean,median

mean(x0AP.t)

median(x0AP.t)

# standard deviation

sd(x0AP.t)

# 95% CI

quantile(x0AP.t, c(0.025, 0.975))

# 4HA accounting for the uncertainty from repeatability and dilution

x0HA.t <- x04HA + rnorm(M, 0, urR.4HA*x0.4HA) + rnorm(M, 0, urD*x0.4HA)

#mean,median

mean(x0HA.t)

median(x0HA.t)

# standard deviation
```

```
sd(x0HA.t)

# 95% CI

quantile(x0HA.t, c(0.025, 0.975))
```

[2] R code for Figure 2

```
library(gridExtra)

library(tidyverse)

library(ggpubr)

library(cowplot)

res <- data.frame(AP = x0AP.t, HA = x0HA.t)

p1 <- ggplot(res, aes(x = AP,
                y = ..density.., )) + labs(x="Concentration (ng/mL)", y = "Density", size=1) +
  geom_histogram(alpha =0.5, fill="#00AFBB") +
  geom_density(alpha=0.2, size=1.2)

p2 <- ggplot(res, aes(x = HA,
                y = ..density..))+ labs(x="Concentration (ng/mL)", y = "Density") +
  geom_histogram(alpha =0.5, fill="#E7B800") +
  geom_density(alpha=0.2, size=1.2)

p3 <- ggarrange(p1, p2, nrow=1, labels=c("AP", "4HA"), label.x=0.8)

p3
```
